# Biomechanical Stress Analysis of Mandibular First Permanent Molar; Restored with Amalgam and Composite Resin: A Computerized Finite Element Study

**DOI:** 10.5005/jp-journals-10005-1047

**Published:** 2010-04-15

**Authors:** Iqbal Musani, AR Prabhakar

**Affiliations:** 1 Professor, Department of Pedodontics and Preventive Dentistry, Bharati Vidyapeeth’s Dental College and Hospital, Pune Maharashtra, India; 2 Professor and Head, Department of Pedodontics and Preventive Dentistry, Bapuji Dental College and Hospital, Davangere Karnataka, India

**Keywords:** Finite element analysis, stress distribution patterns, stress magnitudes, composite resin, amalgam.

## Abstract

Normal mastication with its varying magnitude and direction generates considerable reactionary stresses in teeth and their supporting tissues. The structure of the human tooth and its supporting tissues is a complex assemblage of materials of varied mechanical properties. The finite element method (FEM), a modern technique of numerical stress analysis, has the great advantage of being applicable to solids of irregular geometry and heterogeneous material properties and therefore ideally suited to the examination of structural behavior of teeth. The mandibular first permanent molar is one of the earliest permanent teeth to erupt in the oral cavity and hence most prone to caries. The purpose of the present study was to construct a two-dimensional FE model of the mandibular first permanent molar and its supporting structures, using a FE software called NISA II-Display III, EMRC, USA to study the following:

• To compare stress distributions patterns when a modeled Class I Cavity was restored with dental amalgam and composite resin.

• To compare the stress distributions pattern when the load was applied to different to locations, i.e.: At the mesial cusp tip, and at the center of the occlusal surface.

Both amalgam and composite resin showed similar stress distribution pattern, however, the magnitudes of stresses generated in the tooth restored with composite resin were higher. Thus, amalgam is a better restorative material in distributing stresses.

## INTRODUCTION

Normal mastication with its varying magnitude and direction generates considerable reactionary stresses in teeth and their supporting tissues. Traditional methods of experimental stress analysis, including transmission and reflection two-dimensional photoelasticity, brittle lacquers, and electrical resistance strain gauge techniques have all been used in dental stress analysis.^8^

The structure of the human tooth and its supporting tissues is a complex assemblage of materials of varied mechanical properties. The stiffness of some of the elements are reasonably well-known, those of others are very much in doubt. Stress distribution within a structure is a function of both its shape and the distribution of stiffness within it. Because of the latter, great difficulties would arise in at-tempting a structural model of a tooth. With the photoelastic and other materials that are conveniently available, it is virtually impossible to proportion the tooth model stiffness in the correct manner. The problems associated with direct methods of measuring surface stresses in actual teeth *in vivo* are many and obvious because of the vitality of the tooth, its size and difficulties of access.^8^

Classical methods of mathematical stress analysis are extremely limited in their scope and are inappropriate to dental structures that are of an irregular structural form and complex loading. However, the finite element method, a modern technique of numerical stress analysis, has the great advantage of being applicable to solids of irregular geometry and heterogeneous material properties. It is therefore ideally suited to the examination of the structural behavior of teeth.^8^

The mandibular first molar is amongst the earliest permanent teeth to erupt in the oral cavity and hence is most prone to caries. Dental amalgam has always been the material of choice for restoring a class I lesion on the mandibular first permanent molar. The current awareness amongst patients for esthetics and the demand for tooth colored restorative materials has resulted in pedodontists using composite resin material for posterior restorations.

The present study was conducted at the Department of Pedodontics and Preventive Dentistry, Bapuji Dental College and Hospital in conjunction with the Department of Mechanical Engineering, Bapuji Institute of Engineering and Technology, Davangere. The purpose of this study was to construct a two-dimensional finite element model of the mandibular first permanent molar and its supporing structures, using a finite element software called NISA II-DISPLAY III, EMRC, USA, to study the following:

1. To compare the stress distributions pattern when a modeled class I carious lesion was restored with dental amalgam (Dispersalloy, Johnson and Johnson, USA) and composite resin (Z 100, 3M Dental Products, USA).
2. To compare the stress distribution pattern when the load was applied at different locations, i.e.
  - At the mesial cusp tip
  - At the center of the occlusal surface.
3. To study the effect of different force directions when a 90 kg load was applied to the said locations at an angle:
  - 0° to the long axis of the tooth.
  - 30° to the long axis of the tooth.
  - 60° to the long axis of the tooth.

## INTRODUCTION TO FINITE ELEMENT ANALYSIS

The finite element analysis is a powerful tool for numerical solution of a wide range of engineering problems. The application range from deformation and stress analysis of automotive, aircraft, building and bridge structures to field analysis of heat flow, fluid flow, magnetic flux, seepage and other problems.^4^

Originally introduced as a method for solving structural mechanics problems, FE analysis was quickly recognized as a general procedure of numerical approximation to all physical problems that can be modeled by a differential equation description. FE analysis has also been applied to the description of physical form changes in biological structures particularly in the area of growth and development and restorative dentistry.^3^

## GENERAL PRINCIPLES

FE analysis solves a complex problem by redefining it as the summation of the solutions of series of interrelated simpler problems. The first step is to subdivide (i.e., discretize) the complex geometry into a suitable set of smaller “elements” of “finite” dimensions, which when combined form the “mesh” model of the investigated structure. Each element can adopt a specific geometric shape (i.e., triangle, square, tetrahedron, etc.) with a specific internal strain function. Using these functions and the actual geometry of the element, one can write the equilibrium equations between the external forces acting on the element and the displacements occurring at its corner points or “nodes”. There will be one equation for each degree of freedom for each node of the element. These equations are most conveniently written in matrix form for use in a computer algorithm. From the stiffness matrices of the individual elements, the so-called overall or global stiffness matrix [K] can be assembled for the entire discretized structure. The overall stiffness matrix relates overall forces on the structure to displacements at all the nodes.

{F} = [K] {x} ...(1)

Where, [K] denotes the overall stiffness matrix of the structure, {F} represents the overall force vector which lists the externally applied forces at all the nodes, and {x} symbolizes the displacement at all the nodes.^3^

The global stiffness matrix is then solved for the unknown displacements given the known forces and restraining conditions. This is done by ensuring that the equilibrium and compatibility conditions are satisfied at all nodes in the structure. Whereas, the equilibrium conditions will be satisfied when all forces and moments about a given point equal zero, the compatibility conditions will be ensured if the displacements (i.e., nodal and elemental) within the deformed structure are continuous. These latter conditions thus imply that even though the FE model will yield an approximation of the correct answer, it would be possible to converge on this answer with a less than infinite number of nodes and elements. It is also important to note that equation (1) can be solved only if sufficient number of boundary conditions is introduced. From the displacements at the nodes, the strains in each element can then be calculated, and based on these as well as the material properties, the stresses can be derived.^3^

## PARAMETERS

Basically, four parameters will influence the predictive accuracy of a mechanical FE model. These are:

1. The geometric detail of the object to be modeled
2. The choice of elements type and count,
3. The material properties, and
4. The applied boundary conditions.^3^

### 1. Geometry

The first step in the creation of a finite element model is to represent its geometry in the computer. Depending on the problem to be investigated, the numerical representation of the object under study can be achieved either two-dimensionally (2D) or three-dimensionally (3D) in several ways. In cases of 2D anatomical shapes, their contours are converted into digital format after the tracing of the histo-logical sections or images of any kind.

Although teeth are 3D structures, many of the reviewed tooth models are 2D. Two-dimensional model offer excellent access for pre-and post-processing, and because of the reduced dimensions, computational capacity can be preserved for improvements in element and simulation quality. On the other hand, 3D models, although more realistic with respect to the dimensional properties, are generally more coarse, with elements that are far from their ideal shapes. Moreover, examination of the model is far more difficult. Depending on the investigated structure and boundary conditions, in some instances 2D modeling may be justified as a reasonable or even sensible simplification.^3^

### 2. Element Type and Number

The choice of an appropriate element type will depend on the expected response of the model and thus the accomplishment of the objectives of the analysis. FE analysis offers a wide variety of different element types, which can be categorized by family and topology.^3^

The element family refers to the characteristics of geometry and displacement that the element models. Among the most common families used for typical structural models are one-dimensional beam elements, 2D plane stress and plane strain elements, axisymmetric elements, and 3D shell and solid elements.^3^

Element topology refers to the general shape of the element (e.g., triangular or quadrilateral). The topology also depends on the family of the element (e.g., 2D or 3D).

In general, triangular elements may be considered more suitable than quadrilateral for complex structural models. However, the element with more number of nodes can match the true displacement function more accurately due to a higher number of DOF (i.e., degree of freedom). A DOF represents the liberty of translatory or rotational motion of a particular mode in space.^3^

### 3. Material Properties

The assignment of proper material problems to a FE model is a necessary step to ensure predictive accuracy. Stress-strain relationship in a structure is based on the material properties. These are the Young’s Modulus (or modulus of elasticity) and Poisson’s Ratio.^3^

Material properties in the dental FE analyses are mostly modeled as isotropic and homogenous. Although for dentin this is generally viewed as an acceptable assumption, in the case of enamel it is an oversimplification. Lack of accurate information on anisotropic properties for enamel is cited as justification. The dental pulp has also been included in various dental FE models, but its effect relative to the hard tissues was found to be negligible. The same was established for the omission of cementum.^3^

### 4. Boundary Conditions

The boundary conditions in FE models basically represent the load imposed on the structures under study and their fixation counterparts, the restraints. In addition, they may involve interaction of groups of interconnected finite elements (constraints) or physically separate bodies (contact). The applied boundary conditions are mostly quasi-static.^3^

## APPLICATION OF FINITE ELEMENT IN RESTORATIVE DENTISTRY

Finite element stress analyses in regional dental-related structures have been carried out for two reasons:

1. To study the functions of property and structure in biological teeth.
2. To predict their performance, in particular with respect to mechanical failure. The mechanical behavior of sound teeth is considered the benchmark for restored structures, and as such has been the subject of several stress analyses, alone and in comparison with the restored ones.^3^

### About NISA II/DISPLAY III

The finite element program used in the study was developed by NISA and marketed by engineering mechanic research corporation (IMRC) USA which is one of the most comprehensive and versatile finite element programs in the world today. The NISA family of design/analysis program offers the largest number of finite element application program which are completely integrated through interactive graphical interface called “DISPLAY III”. This integration is a powerful tool in analyzing design alternatives for almost any combination of load environment till an optimum design is reached.^4^

DISPLAY III is a three-dimensional interactive color graphics program for geometric and finite element modeling and result postprocessing. This program is menu driven and modeling is achieved with the help of a mouse.^4^

## MATERIALS AND METHODS

The three primary considerations in the development of the finite element model of the restored tooth and its supporting tissues are:

### 1. Tooth Geometrics

**Fig. 1:**
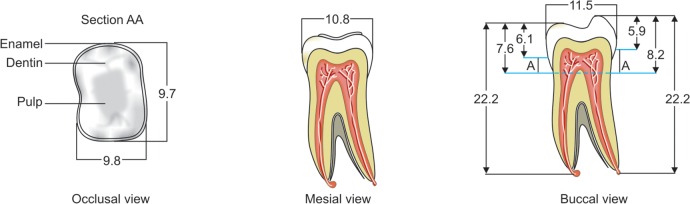
Dimensions of the mandibular first permanent molar

**Fig. 2:**
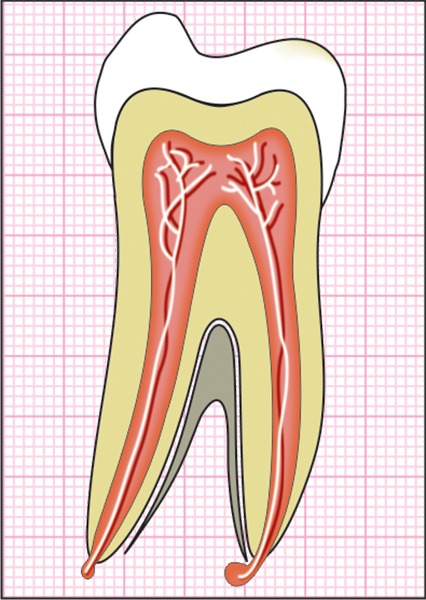
Graphical representation of the mandibular first permanent molar

**Fig. 3:**
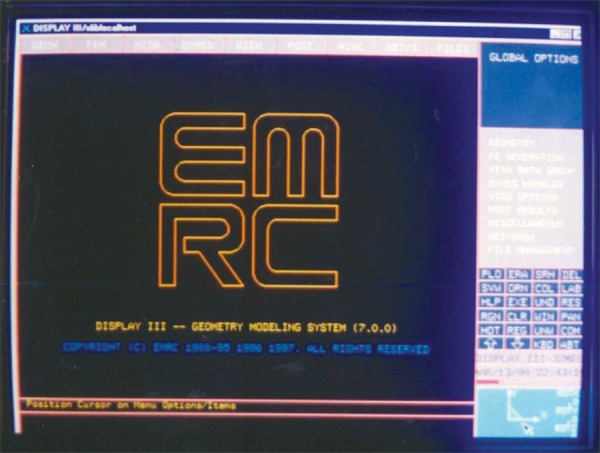
EMRC - Software used for the study

**Fig. 4:**
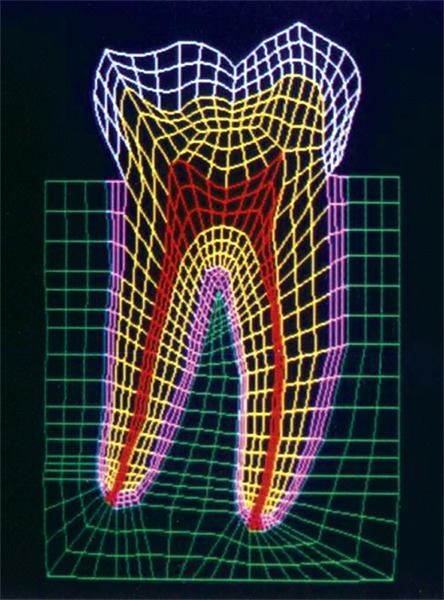
Finite element model of the tooth with its supporting tissues

The mesio-distal section of the mandibular first permanent molar and its supporting tissue as reported by Rubin et al., was used for the model construction (Fig. 1). The tooth outlines were traced on a graph paper and the (x, y) co-ordinates were found out (Fig. 2). This data was transferred to the Display III software and a geometric modeling was done (Fig. 3). Care was taken to approximate the contours and morphology of the tooth. The two-dimensional tooth and its supporting tissues were divided into 1053 element areas and 1102 nodes. Quadrilateral element type was used (Fig. 4).

**Fig. 5:**
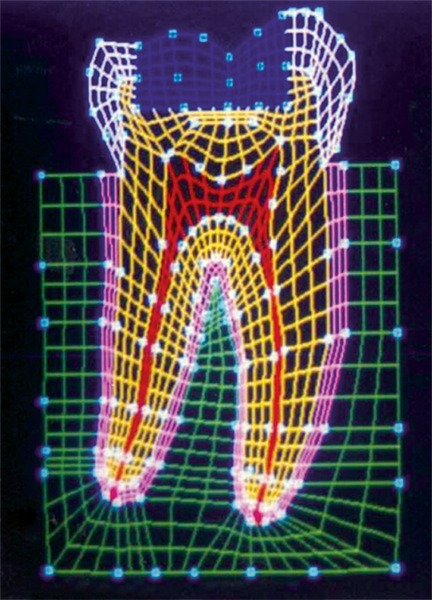
Finite element model of the tooth with a class I restoration

A cavity of 7.5 mm mesio-distal width and 0.5 mm depth in to dentin was incorporated in the model (Fig. 5). The cavity dimensions are similar to the one used by Shu-Min Zhou et al.^7^ This cavity was restored first with amalgam and then composite resin, i.e. the data of material properties was changed to study the behavior of the restored tooth for the same cavity. This was done to standardize the dimensions and design of the cavity and study the comparative stress distributions pattern under identical loading conditions to evaluate the efficacy of the two restorative materials used.

### 2. Material Properties

The modulus of elasticity and Poisson’s ratio of tooth tissues, periodontal ligament, alveolar bone and restorative materials have been previously reported in the literature, and shown in the table. These were assigned appropriately to the model.

**Table:** 

*Sr.* * no.*		*Tissue/restorative* *material*		*Young’s* *modulus of* *elasticity (GPa)*		*Poisson’s* *ratio*	
1.		Enamel		84.1		0.33	
2.		Dentin		18.0		0.31	
3.		Pulp		0.002		0.45	
4.		PDL		0.00345		0.45	
5.		Alveolar bone		13.8		0.30	
6.		Dental amalgam (Dispersalloy,					
		Johnson and Johnson, USA)		48.3		0.35	
7.		Composite resin (Z 100, 3M					
		Dental products,USA)		20.0		0.24	

### 3. Masticatory Forces

The locations, magnitudes and directions of the tooth loading due to masticatory forces vary dramatically for different individuals. Maximum values for individuals with normal dentition in the molar region may range from 45 to 90 kg.^5^

**Fig. 6:**
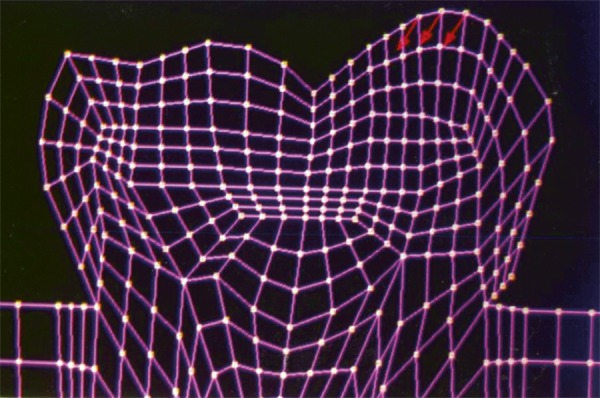
Load applied at the mesial cusp tip

**Fig. 7:**
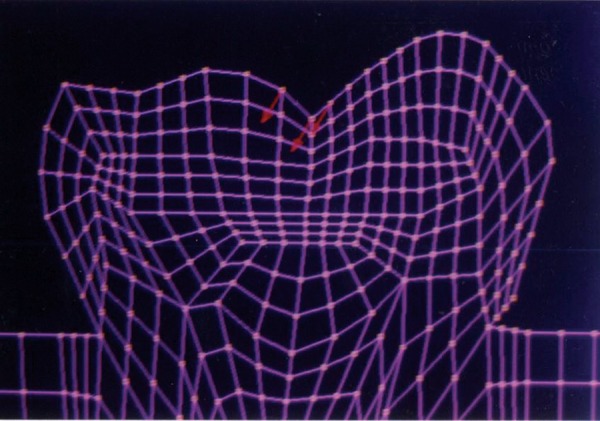
Load applied at the center of the occlusal surface

A distributed load of 90 kg was applied to the:

1. Mesial cusp tip (Fig. 6)
2. Center of the occlusal surface (Fig. 7)

**Fig. 8:**
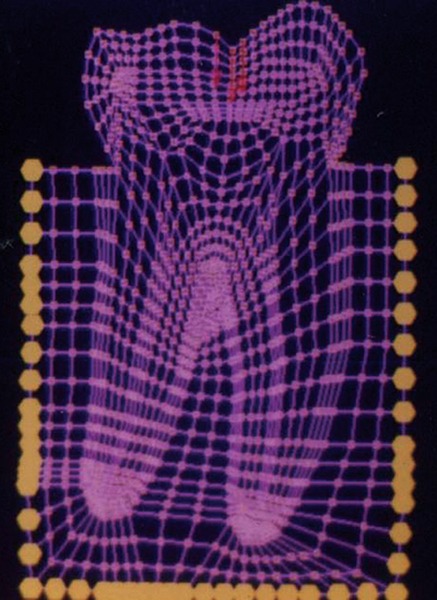
Load applied at 0° to the long axis of the tooth

**Fig. 9:**
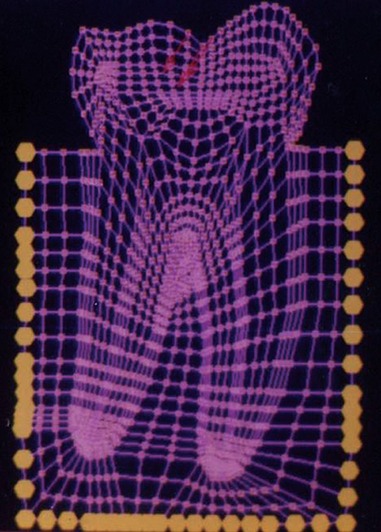
Load applied at 30° to the long axis of the tooth

**Fig. 10:**
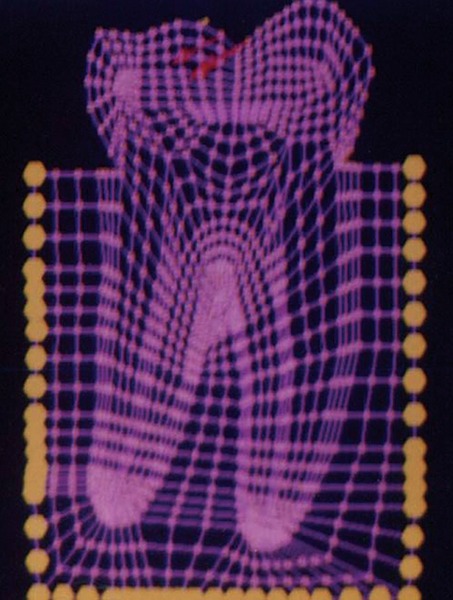
Load applied at 60° to the long axis of the tooth

In the following directions:

- 0° to long axis of the tooth (Fig. 8).
- 30° to long axis of the tooth (Fig. 9).
- 60° to long axis of the tooth (Fig. 10).

A study comparing the stress distributions pattern of normal tooth with that of a tooth with Class I occlusal restoration using similar loading parameters was carried out by Shu-Min Zhou et al (1989).^7^

In the present study, comparison has been made between the stress distributions for Class I occlusal restoration with amalgam and composite resin.

**Fig. 11:**
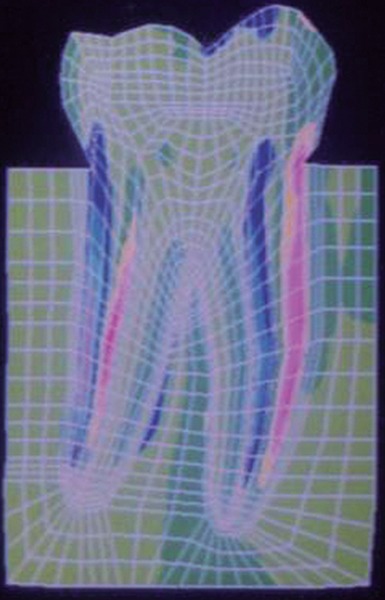
SYY stresses

**Fig. 12:**
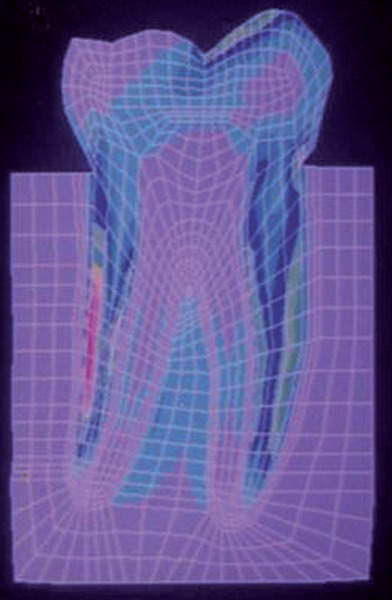
Von Mises stress

The stresses studied were:

- SY-Stresses (Fig. 11), i.e. stresses in the directions of occlusal loading.
- Von-Mises stresses (Fig. 12), i.e. total cumulative stresses.

## RESULTS

**Fig. 13:**
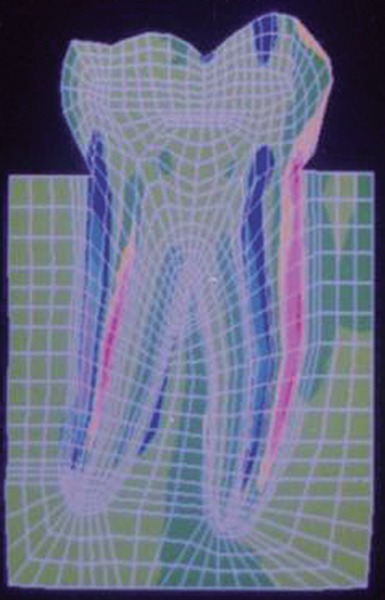
SYY stresses–location of loading: mesial cusp tip at 60° to the long axis for composite restoration

**Fig. 14:**
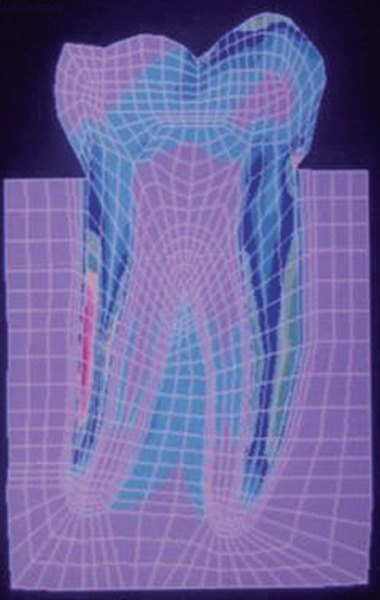
Von Mises stress–location of loading: mesial cusp tip at 60° to the long axis for composite resin restoration

**Fig. 15:**
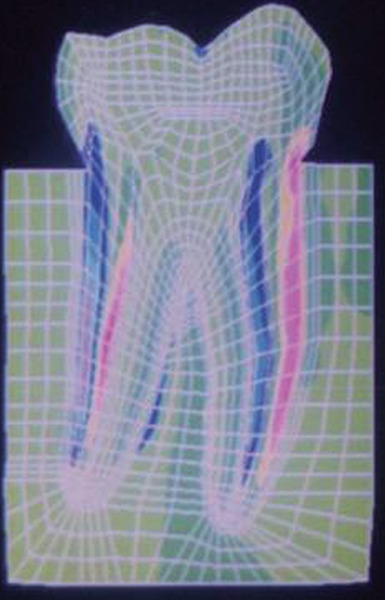
SYY stresses–location of loading: center of occlusal surface at 60° to the long axis for amalgam restoration

**Fig. 16:**
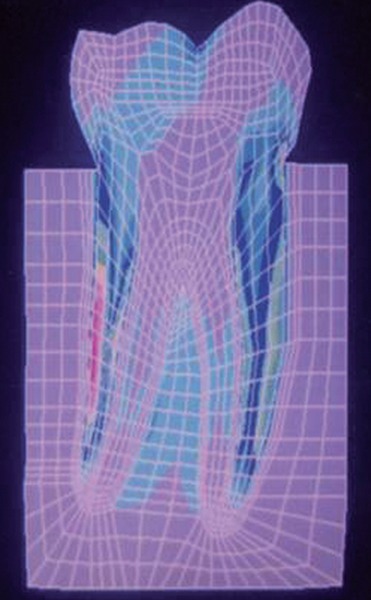
Von Mises stress–location of loading: center of oc-clusal surface at 60° to the long axis for amalgam restoration

**Fig. 17:**
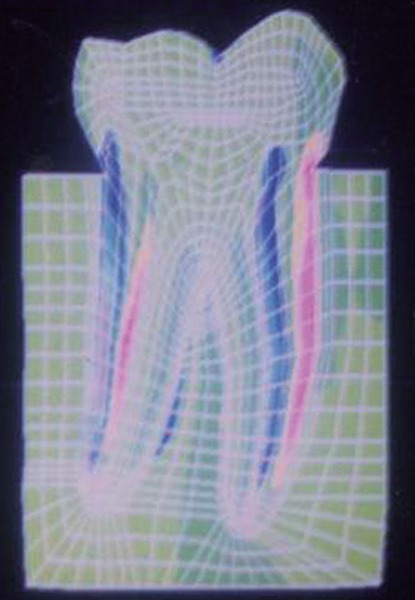
SYY stresses–location of loading: center of occlusal surface at 60° to the long axis for composite resin restoration

**Fig. 18:**
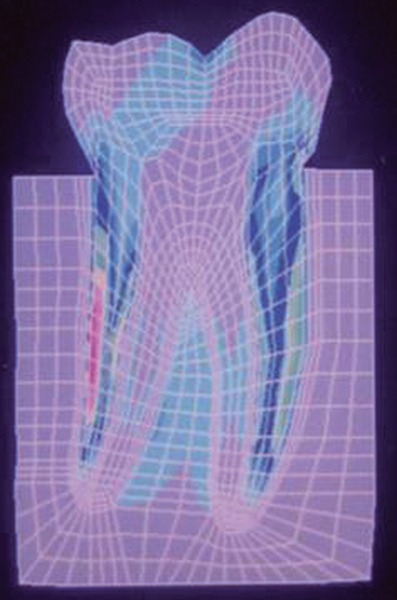
Von Mises stress–location of loading: Center of occlusal surface at 60° to the long axis for composite resin restoration

The stress distributions patterns were analyzed and results tabulated and graphically represented. Since the cervical thirds of the crown showed an increased magnitude of stresses, a detailed analysis of this region was carried out. Since the maximum stresses were seen at load application of 60°, all photographs of this loading condition are presented here (Figs 11 to 18).

## MAXIMUM STRESS VALUES (MPA) IN THE CERVICAL THIRDS OF THE CROWN AND MAXIMUM VON MISES STRESS VALUES (MPa) WHEN THE LOAD WAS APPLIED TO THE MESIAL CUSP TIP (TABLES 1 TO 3)

**Table 1:** **Table 1:** Load applied at 0° to the long axis

		*Amalgam* * restoration*		*Composite* * restoration*	
Compressive stress		– 14.38		– 14.95	
Tensile stress		5.69		8.24	
Von Mises stress		21.60		24.19	

**Table 2:** **Table 2:** Load applied at 30° to the long axis

		*Amalgam* * restoration*		*Composite* * restoration*	
Compressive stress		– 3.56		– 3.65	
Tensile stress		3.55		3.57	
Von Mises stress		6.72		6.73	

**Table 3:** **Table 3:** Load applied at 60° to the long axis

		*Amalgam* * restoration*		*Composite* * restoration*	
Compressive stress		– 6.63		– 9.96	
Tensile stress		8.50		13.50	
Von Mises stress		15.93		15.87	

## MAXIMUM STRESS VALUES (MPA) IN THE CERVICAL THIRDS OF THE CROWN AND MAXI-MUM VON MISES STRESS VALUES (MPa) WHEN THE LOAD WAS APPLIED TO THE CENTER OF OCCLUSAL SURFACE (TABLES 4 TO 6)

**Table 4:** **Table 4:** Load applied at 0° to the long axis

		*Amalgam* * restoration*		*Composite* * restoration*	
Compressive stress		– 3.54		– 4.52	
Tensile stress		2.48		2.67	
Von Mises stress		3.68		4.46	

**Table 5:** **Table 5:** Load applied at 30° to the long axis

		*Amalgam* * restoration*		*Composite* * restoration*	
Compressive stress		– 5.92		– 6.84	
Tensile stress		4.84		7.02	
Von Mises stress		8.64		9.37	

**Table 6:** **Table 6:** Load applied at 60° to the long axis

		*Amalgam* * restoration*		*Composite* * restoration*	
Compressive stress		– 6.64		– 6.65	
Tensile stress		10.23		13.39	
Von Mises stress		14.20		15.96	

## DISCUSSION

The energy of the bite is absorbed by the food bolus during mastication, as well as by the teeth, periodontal ligament, and bone. Nevertheless, the design of the tooth is an engineering marvel in that the tooth is generally able to absorb such static as well as dynamic (impact) energies. The modulus of resilience of dentin is greater than that of enamel and thus is better able to absorb impact energy. Enamel is a brittle substance with a comparatively high modulus of elasticity, a low proportional limit in tension, and a low modulus of resilience. However, although it is supported by dentin with significant ability to deform elastically, teeth seldom fracture under normal occlusion.^5^

Normal tooth structure transfers external biting loads through enamel in to the dentin as compression. The concentrated external loads are distributed over a large internal volume of tooth structure and thus local stresses are lower. During this process a small amount of dentin deformation may occur which results in tooth flexure. A restored tooth tends to transfer stresses differently than an intact tooth. Any force on the restoration produces complex stresses along the tooth-restoration interface. Once enamel is no longer continuous, its resistance is much lower. Once in dentin, the stresses are resolved in a manner similar to a normal tooth.^1^

Any finite element model relies on several assumptions.^6^

Tooth materials and restorative materials in this model were assumed to be homogenous, isotropic, elastic and functioning in a linear fashion (Hooke’s law).^2^

As assumed by Farah et al.,^2^ in this study too the pulpal floor of the cavity preparation was assumed to be placed on sound dentin and it was further assumed that the restorative material was fixed to the cavity wall or in other words to have good retention and adherence to the dentin and the enamel. The bottom of the model was assumed to be fixed to prevent rigid body displacement.^2^

While a healthy tooth is in function, the crown is mainly under considerable compressive stress, only a few parts of the crown undergo tensile stress, and the magnitude of this stress is only about one seventh that of the compres-sive stress. This corresponds to the material properties of the tooth, i.e. the tensile strength is about one-seventh of the compressive strength.^7^ However, in a restored tooth a significant increase in the magnitude of tensile stresses was seen in this study.

The results of this study showed that the highest stresses are borne by the root, followed by the cervical thirds of the crown, followed by the restoration-tooth interface. The pulp chamber and root canals bear negligible stresses. The supporting bone bears minimal stresses. The mesial cusp tip and the center of occlusal surface bear compressive stresses because of the point of application of load.

The junction between the clinical root and the clinical crown bears tremendous stresses. There is compression on the occluding side and tension on the noncontacting side. As stated by Yettram et al,^9^ this could be because the reacted forces have to flow in to and through the thin wedge of tissue for them to be transmitted in to the root of the tooth and subsequently in to the supporting alveolus.

Another observation made from this study and that reported by Yettram et al,^9^ is that because the enamel demonstrated greater stiffness than the dentin, the enamel absorbed most of the occlusal force and so displayed higher stresses than those absorbed in the dentin. The results in this study showed higher localized stresses in enamel reaching its peak in the cervical region and lower and more evenly distributed stresses in the dentin.

The results also indicate that the chances of fracture or failure of the restoration at the tooth-restoration interface are remote. However, the model treated here assumes the existence of very ideal conditions at and within the cavity preparation, (that is homogeneity of restorative material, application and distribution of the load, complete retention at the cavity wall and above all the lack of clinically introduced variables).

When the molar was restored with amalgam and subjected to loading at the mesial cusp tip at different angles, the maximum stress values were seen at 0° loading. At 30° loading the values were lower and these again increased at 60°. At 0° and 30° loading the mesial aspect of the cervical thirds of the crown showed compression and the distal aspect showed tension. However, at 60° loading the mesial aspect showed tension and distal aspect compression.

A similar observation was made when the molar was restored with composite resin and loaded similarly at the mesial cup tip. A point to be highlighted here is that the magnitudes of stresses generated in the tooth restored with composite resin were higher than that for the tooth restored with amalgam.

When the molar was restored with amalgam and loaded at the center of the occlusal surface at different angles, the stress magnitudes increased as the angle of application of load increased from 0° through 60°. A similar observation was made when the tooth was restored with composite resin. The stresses generated again were greater in magnitude for composite resin than amalgam.

In general lower magnitudes of stresses were seen when the tooth was loaded at the center of the occlusal surface than when it was loaded at the mesial cusp tip for all angulations of loading.

The cervical thirds of the crown showed tensile stresses on the mesial aspect and compressive stresses on the distal aspect when loaded at the center of the occlusal surface for all the three loading angles and also when the tooth was loaded at 60° at the mesial cusp tip. However, at 0° and 30° loading at the mesial cusp tip it showed a reversal, that is compressive stresses on the mesial aspect and tensile on the distal aspect.

In general the stress distributions pattern was similar for both amalgam and composite restorations. However, the magnitude of stresses generated were considerably higher for composite resin than for dental amalgam. This is attributed to the lower modulus of elasticity of the composite resin than dental amalgam.

The main advantage of the model used in this study is that the magnitude, direction and location of the load are reproducible. Since all other variables are controlled, the results depend strictly on the mechanical properties of the restorative materials and reflect the behavior of the materials.

## SUMMARY AND CONCLUSIONS

A two-dimensional finite element model of a mandibular first permanent molar was created on NISA - II / DISPLAY III for this study. A comparative stress analysis was carried out for a modeled Class I lesion restored with dental amalgam and composite resin.

Analyses were carried out to study the effects of the change in the location of application of load and also for the change in angulation of the applied load. The following conclusions were draws:

1. The best stress distributions pattern with minimal stress values were seen when the tooth was restored with dental amalgam and loaded at the center of the occlusal surface axially.
2. As the force angle increased, the stress values increase, high magnitudes of stresses were seen in the cervical thirds of the crown.
3. When the tooth was loaded at the mesial cusp tip, comparatively higher magnitudes of both tensile and compressive stresses were seen in the cervical third of the crown thus producing a potentially damaging environment for the remaining tooth structure, which would lead ultimately to fracture.
4. Both amalgam and composite resin showed similar stress distribution pattern, however, the magnitudes of stresses generated in the tooth restored with composite resin were higher. Thus, amalgam is a better restorative material in distributing stresses.

The finite element method is the nearest possible method available today to simulate the oral cavity *in vitro.* It is a numerical method for addressing mechanical problems and therefore, is a powerful contemporary research tool. FE analysis provides a precise insight into the complex mechanical behavior of restored teeth affected by stress fields which are difficult to assess otherwise. Of particular importance is the possibility of examining the various parameters. The use of these theoretical engineering methods will certainly give answers to problems in restorative dentistry. Thus the results are practical and applicable, of clinical significance and reference value and give direction to experimental and clinical research.
